# Targeted therapy of glioblastoma stem-like cells and tumor non-stem cells using cetuximab-conjugated iron-oxide nanoparticles

**DOI:** 10.18632/oncotarget.3554

**Published:** 2015-03-12

**Authors:** Milota Kaluzova, Alexandros Bouras, Revaz Machaidze, Costas G. Hadjipanayis

**Affiliations:** ^1^ Brain Tumor Nanotechnology Laboratory, Department of Neurosurgery, Emory, University, School of Medicine, Winship Cancer Institute of Emory University, Atlanta, GA, USA; ^2^ Winship Brain Tumor Center of Emory University, Winship Cancer Institute of Emory University, Atlanta, GA, USA; ^3^ Present address: Brigham and Women's Hospital, Neurosurgery, Boston, MA, USA

**Keywords:** glioblastoma stem-like cells (GSCs), iron-oxide nanoparticles (IONPs), cetuximab, magnetic resonance imaging (MRI), convection-enhanced delivery (CED)

## Abstract

Malignant gliomas remain aggressive and lethal primary brain tumors in adults. The epidermal growth factor receptor (EGFR) is frequently overexpressed in the most common malignant glioma, glioblastoma (GBM), and represents an important therapeutic target. GBM stem-like cells (GSCs) present in tumors are felt to be highly tumorigenic and responsible for tumor recurrence. Multifunctional magnetic iron-oxide nanoparticles (IONPs) can be directly imaged by magnetic resonance imaging (MRI) and designed to therapeutically target cancer cells. The targeting effects of IONPs conjugated to the EGFR inhibitor, cetuximab (cetuximab-IONPs), were determined with EGFR- and EGFRvIII-expressing human GBM neurospheres and GSCs. Transmission electron microscopy revealed cetuximab-IONP GBM cell binding and internalization. Fluorescence microscopy and Prussian blue staining showed increased uptake of cetuximab-IONPs by EGFR- as well as EGFRvIII-expressing GSCs and neurospheres in comparison to cetuximab or free IONPs. Treatment with cetuximab-IONPs resulted in a significant antitumor effect that was greater than with cetuximab alone due to more efficient, CD133-independent cellular targeting and uptake, EGFR signaling alterations, EGFR internalization, and apoptosis induction in EGFR-expressing GSCs and neurospheres. A significant increase in survival was found after cetuximab-IONP convection-enhanced delivery treatment of 3 intracranial rodent GBM models employing human EGFR-expressing GBM xenografts.

## INTRODUCTION

Malignant gliomas remain a deadly form of brain cancer with a poor prognosis despite surgery, chemotherapy, and ionizing radiation [1]. The most common malignant glioma, known as glioblastoma (GBM), is characterized by necrosis, hypoxia, and extensive angiogenesis [2]. Highly invasive GBM cells are known to infiltrate the surrounding normal brain, making complete resection impossible [3, 4]. There is a clear need for more effective strategies for the treatment of GBM.

GBM stem-like cells (GSCs) represent a subpopulation of the tumor which is responsible for tumor initiation, progression, and re-growth after chemoradiation [5, 6], as well as supporting tumor vessel growth and function [7]. GSCs are considered a relevant target for therapy of GBM [8]. Expression of various cell surface markers on GSCs have been reported, including CD133 [9], SSEA1 [10], and CD44 [11]. CD133-positive populations, isolated from human GBM surgical samples, have been shown to initiate the growth of GBM tumors *in vivo* that recapitulate human tumors [9]. CD133-positive human GBM cells secrete a high level of vascular endothelial growth factor (VEGF) which can contribute to their tumor-initiating capacity [12].

The epidermal growth factor receptor (EGFR), including the EGFRvIII deletion mutant, is overexpressed in the majority of GBM tumors and represents a major target for treatment of these tumors [13, 14]. The Cancer Genome Atlas (TCGA) has shown that the high level of EGFR expression correlates with EGFR gene amplification [15, 16] and indicates a poor prognosis in GBM patients [17]. EGFR has been used for targeting GSCs previously [18, 19]. Cetuximab (Erbitux; ImClone Inc.), a 152 kDa chimeric monoclonal antibody of the immunoglobulin G1 subclass that binds to the extracellular domain of the human EGFR [20], has been used to treat GBM [21]. Targeting of both the wild-type (wt) EGFR and the EGFRvIII deletion mutant is possible with cetuximab [22, 23]. Cetuximab was found to have an inhibitory effect against GBM cell lines and *in vivo* when systemically administered in xenograft mouse models [21, 22, 24, 25]. The use of cetuximab for GBM patients has been limited due to its larger size and difficulty crossing the blood brain barrier (BBB) similar to other anti-EGFR antibodies [23, 26-28]. Cetuximab has also been evaluated preclinically in a rodent glioma model alone [29], as a delivery agent for methotrexate [30], and boron neutron capture therapy after intratumoral convection-enhanced delivery (CED) [31].

Magnetic iron-oxide nanoparticles (IONPs) are becoming an increasingly versatile and potent tool in modern medicine. They can be used for clinical detection by direct magnetic resonance imaging (MRI) due to their strong hypointense T_2_ weighted signal (T2WI) [32]. They also offer the ability to attach tumor-specific biomolecules to their biocompatible surface for tumor targeting [33-35]. To reduce nonspecific interactions of IONPs with cells, a polyethylene glycol (PEG) coating can be used to modify the nanoparticle surface [36, 37]. CED is a method for delivering therapeutic agents directly to brain tumors by avoiding the BBB. CED permits distribution of molecules through the brain interstitial spaces by a pressure gradient applied through a catheter implanted in the brain [38]. Direct delivery into the brain can provide higher concentrations of therapeutic agents in and around brain tumors while minimizing systemic toxic effects.

The main objective of this study was to investigate the therapeutic targeting effect of cetuximab-IONPs against EGFR- and EGFRvIII-expressing GSCs in addition to GBM tumor non-stem cells. Compared to cetuximab alone, our data support the findings of increased binding by cetuximab-IONPs to EGFR- and EGFRvIII-expressing GBM cells, including GSCs. Greater binding of cetuximab-IONPs and EGFR inhibition results in downstream EGFR cell signaling aberrations. We have also found greater intracellular presence of cetuximab-IONPs and greater translocation of EGFR into the cytoplasm, specifically the cytoskeletal fraction of cells. In combination, greater binding to EGFR, inhibition of EGFR, as well as internalization of the cetuximab-IONPs and EGFR trigger apoptosis in human EGFR-expressing GBM cells including GSCs. The targeted therapy of cetuximab-IONPs with CED *in vivo* revealed a significant therapeutic effect in three different orthotopic mouse models of human GBM.

## RESULTS

### EGFR and stem cell markers expression in human GSCs-containing GBM neurospheres

GBM neurospheres are pathologically relevant models that stably maintain genomic changes of the primary tumor, exhibit stem-like tumor properties, and recapitulate the invasive behavior of GBM [39]. Early passage neurospheres derived from fresh human surgical specimens of eight GBM patients were analyzed for wtEGFR overexpression or expression of the EGFRvIII deletion mutant. Western blotting confirmed that, relative to normal astrocytes, all neurosphere cultures express higher levels of wtEGFR and that these levels varied in the neurosphere set: N08-30 displayed strong, N08-74, N08-1002, N09-30, N09-33, N09-20 and N09-21 intermediate, and N09-32 weak EGFR expression. Only the N08-30 neurospheres were positive for both wtEGFR and the EGFRvIII mutant (Supplementary Figure S1A, top). The ability of GBM neurospheres to maintain wtEGFR expression after *in vitro* passaging was confirmed by higher expression of wtEGFR in GBM neurospheres compared to normal human astrocytes (Supplementary Figure S1A, bottom) and neural human progenitor cells (NHPC) (Supplementary Figure S1B). In all other experiments, however, neurospheres in early passage were used. Neurospheres N08-74, N08-1002, N08-30, N09-30, N09-32, and N09-33 were positive for the stem cell marker CD133, N08-30 and N09-32 displaying very strong expression (Supplementary Figure S1C). All neurospheres were positive for stem cell markers nestin, Nanog, and Sox-2, except for N08-21, which was positive only for nestin and Nanog. N09-20 had a low level of Sox2 (Supplementary Figure S1D). Expression of the stem cell marker CD133 in GBM neurospheres was further characterized by Flow cytometry (FACS) (Supplementary Figure S2A).

For further studies, we chose the EGFRvIII/wtEGFR-positive N08-30 and the wtEGFR-positive N08-74 and N08-1002 neurospheres.

### Multilineage differentiation and tumorigenicity of human GBM neurospheres

N08-74, N08-30, and N08-1002 neurospheres formed invasive tumors in athymic nude mice brains within 4-11 months after implantation as confirmed by MRI and histological examination (Supplementary Figure S2B). All neurospheres tested showed multi-lineage differentiation. When grown in neurobasal medium supplemented with 10% FCS, neurospheres became positive for glial (GFAP) and neuronal (Tuj 1) differentiation markers (Supplementary Figure S2C). At the same time, no or significantly decreased expression of the neuronal marker Olig 2 and the stem cell marker CD133 was observed (Supplementary Figure S2D).

### Preparation, physicochemical characterization of bioconjugated IONPs, and their cellular uptake

**Figure 1:**
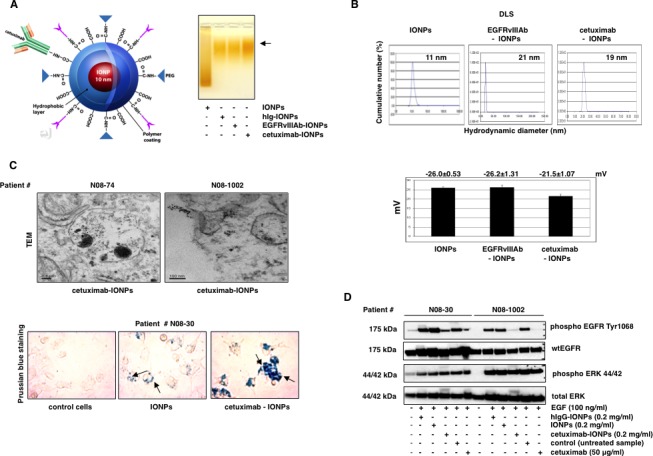
Physicochemical characterization and *in vitro* uptake of the cetuximab-IONPs (A, left) Illustration of amphiphilic triblock copolymer-coated IONPs conjugated to cetuximab. (A, right) Confirmation of conjugation of IONPs to cetuximab, EGFRvIIIAb, and a human IgG by mobility shift (black arrow) in 1% agarose gel. (B, top) Dynamic light scattering (DLS) and hydrodynamic diameter of IONPs, cetuximab-IONPs, and EGFRvIIIAb-IONPs. (B, bottom) Zeta potential of IONPs, EGFRvIIIAb-IONPs, and cetuximab-IONPs. (C, top) Transmission electron microscopy (TEM) studies of cell binding and internalization of cetuximab-IONPs into lysosomes of human GBM neurospheres N08-74 and N08-1002 (magnification 10,000x). (C, bottom) Prussian blue staining of control (no treatment), IONPs, and cetuximab-IONPs internalized by human GBM neurospheres N08-30 (representative slides are shown). Neurospheres were allowed to attach to cell culture dish after treatment in neurobasal media. Nanoparticles are indicated by black arrows, magnification 40x. Cetuximab-IONPs showed maximal uptake. (D) Effect of cetuximab-IONPs on phosphorylation of EGFR after activation with EGF. Human GBM neurospheres N08-30 and N08-1002 were starved for 24 hs, pretreated with cetuximab-IONPs, IONPs, hIgG-IONPs, or cetuximab for 3 hs, followed by activation with 100 ng/ml EGF for 15 min. Western blotting with phospho-EGFR Y1068 antibody shows decreased activation of EGFR in the presence of cetuximab-IONPs. Total ERK 44/42 was used as an internal control.

A schematic diagram of cetuximab-IONPs is shown in Figure 1A, left. Covalent antibody conjugation to amphiphilic triblock copolymer-coated IONPs (PEG MW 2000) [40] (kit by Ocean Nanotech Inc., Little Rock, AK) was confirmed by mobility shift in agarose gel electrophoresis (Figure 1A, right). Briefly, for antibody conjugation, activation of the carboxyl groups on the IONPs was performed in an activation buffer containing ethyl dimethylaminopropyl carbodiimide (EDC) and NHS (sulfo-N-hydroxysuccinimide). Conjugation of IONPs to amino groups of cetuximab, human IgG, and EGFRvIII antibodies was carried out at an approximate 1: 1 (IONPs: antibody) molar ratio. Dynamic light scattering (DLS) analysis revealed that the mean diameters of the cetuximab-IONPs, EGFRvIIIAb-IONPs, and free IONPs were 19, 21, and 11 nm, respectively (Figure 1B, top). The zeta potentials of the cetuximab-IONPs, EGFRvIIIAb-IONPs, and free IONPs were −21.5±1.07, −26.2±1.31, and −26.0±0.53 mV, respectively (Figure 1B, bottom). To evaluate the cellular uptake of the cetuximab-IONPs, we performed transmission electron microscopy (TEM) and Prussian blue staining after treatment of cells. TEM revealed uptake of cetuximab-IONPs by GBM neurospheres N08-74 and N08-1002. Nanoparticles were found freely within the cytosol and in endosomes, suggesting endocytosis of the cetuximab-IONPs (Figure 1C, top). To assess the targeting effect by cell binding, we compared Prussian blue staining of GBM neurospheres N08-30 incubated with IONPs and cetuximab-IONPs for 24 hs. Data in Figure 1C, bottom confirm the highest uptake of cetuximab-IONPs, suggesting improved internalization of cetuximab-IONPs compared to IONPs. Markedly decreased phosphorylation of Y1068, one of the major autophosphorylation sites on EGFR, in cetuximab-IONP-treated N08-30 and N08-1002 neurospheres (Figure 1D) confirm the biological activity of cetuximab-IONPs on EGFR signaling in GBM neurospheres.

### Cytotoxic effect of cetuximab-IONPs in human GBM neurospheres *in vitro*

**Figure 2:**
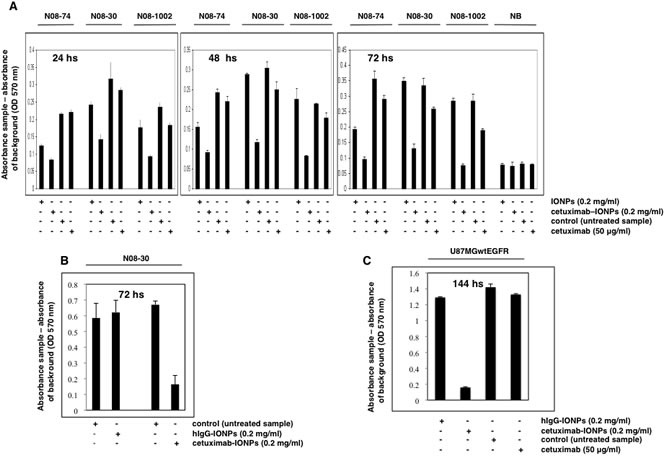
Cytotoxicity of cetuximab-IONPs in human GBM neurospheres and U87MGwtEGFR GBM cell line and quantification by an MTT assay (A) Neurospheres N08-74, N08-30, and N08-1002 (3×10^4^ cells per well) and normal brain cells (NB, 5×10^3^) were treated with free IONPs (0.2 mg/ml), cetuximab-IONPs (0.2 mg/ml), control vehicle, or cetuximab alone (50 μg/ml) and MTT assay was performed after 24, 48, and 72 hs (GBM neurospheres) or 72 hs (normal brain cells). A significant decrease in cell survival was observed in GBM neurospheres treated with cetuximab-IONPs for 72 hs (*P*<0.001). No cytotoxicity was observed in normal brain cells after 72 hs. (B) Neurospheres N08-30 were treated with 0.2 mg/ml cetuximab-IONPs or IgG-IONPs for 72 hs when an MTT assay was performed. Only cetuximab-IONPs displayed increased cytotoxicity (*P*<0.001). (C) U87MGwtEGFR cells (5×10^3^) were treated with hIgG-IONPs (0.2 mg/ml), cetuximab-IONPs (0.2 mg/ml), control vehicle, or cetuximab alone (50 μg/ml) for 144 hs. A significant decrease in cell survival was found in U87MGwtEGFR GBM cell treated with the cetuximab-IONPs (*P*<0.001). In all experiments, neurospheres and other cells were used in early passage.

Cetuximab was previously reported to elicit significant cytotoxicity and increased apoptosis in GBM cell lines with EGFR amplification [21]. We tested the effect of cetuximab-IONPs on the growth of GBM neurospheres with varying levels of EGFR expression. Neurospheres N08-74, N08-30, and N08-1002 were incubated with free IONPs (0.2 mg/ml), cetuximab-IONPs (0.2 mg/ml), and cetuximab (50 μg/ml) alone for 24, 48, and 72 hs (50 μg/ml cetuximab was used since in the conjugation reaction we used 1: 1 molar ratio, equivalent to 4 μg IONPs: 1 μg cetuximab). Cells from adult normal human brain (NB) were used as a control. A cell proliferation and viability assay was performed after each time point. In this assay, the absorbance of control (untreated neurospheres) cells is considered as 100% of live cells and the absorbance of treated cells is proportionate to that of the control cells. Absorbance was corrected by subtracting the background (IONPs in media, or media only, respectively). Cetuximab-IONPs significantly decreased cell survival in all neurospheres tested, most prominently after 72 h treatment *(P*<0.001*)* whereas cetuximab alone had a modest effect on neurosphere cell viability. Due to nonspecific uptake, free IONPs also compromised cell survival in N08-74 neurospheres, but much less efficiently than cetuximab-IONPs. None of the treatments was toxic to human astrocytes (data not shown) and NB cells after 72 hs (Figure 2A). In contrast to hIgG-IONPs, cetuximab-IONPs decreased viability of N08-30 GBM neurospheres (Figure 2B) and in a dose-dependent manner (data not shown). These results confirm that the conjugation of cetuximab to IONPs is crucial for enhanced cytotoxicity of cetuximab-IONPs against all GBM neurospheres. As an additional control, we used the GBM cell line U87MGwtEGFR (overexpressing wtEGFR). Treatment with hIgG-IONPs, cetuximab-IONPs, control vehicle, or cetuximab for 144 hs resulted in significantly decreased survival only in cells treated with cetuximab-IONPs (Figure 2C). In the parental cell line U87MG with low basal wtEGFR expression, the cytotoxic effect of cetuximab-IONPs was about 6x lower than in U87MGwtEGFR (data not shown). Together, these results indicate a significant and selective EGFR-dependent cytotoxic effect of cetuximab-IONPs on the growth of GBM neurospheres and cell lines and no toxicity with normal brain cells.

### Cetuximab-IONPs induce apoptosis and wtEGFR internalization in human GBM neurospheres

**Figure 3:**
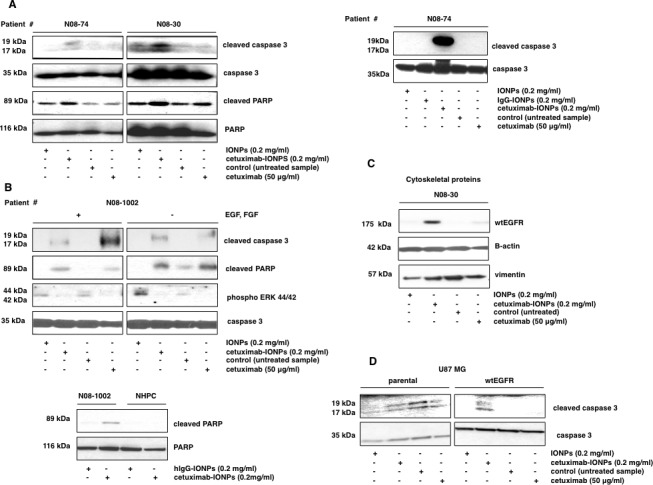
Apoptosis in human GBM neurospheres containing GSCs treated with cetuximab-IONPs **Transport of EGFR to the cytoskeletal structures**. Neurospheres were treated with free IONPs (0.2 mg/ml), cetuximab-IONPs (0.2 mg/ml), control vehicle, or cetuximab alone (50 μg/ml) and expression of apoptotic proteins was evaluated by Western blotting. Elevated levels of cleaved caspase 3 and cleaved PARP were found in neurospheres N08-74 and N08-30 after treatment with cetuximab-IONPs for 3 (A, left) and in neurospheres N08-74 for 14 hs (A, right). Treatment with cetuximab-IONPs was most effective in inducing cleavage of caspase 3 and PARP although some caspase 3 cleavage was also induced by free IONPs in N08-30. In neurospheres N08-1002, induction of caspase 3 and PARP cleavage, and decreased phosphorylation of ERK 44/42 was found after 3 h treatment with cetuximab-IONPs and cetuximab alone, both in the presence and absence of EGF and FGF, caspase 3 was used as a control (B, top). Treatment with cetuximab-IONPs (but not the control conjugated antibody) increased cleavage of PARP in neurospheres N08-1002 whereas no cleavage was observed in NHPC (B, bottom). (C) N08-30 neurospheres were treated as above for 5 hs, lysates were subcellularly fractionated, and analyzed by Western blotting. Elevated levels of wtEGFR were found in the cytoskeletal fraction after cells were treated with cetuximab-IONPs. (D) U87MG and U87MGwtEGFR human GBM cell lines were treated with free IONPs, cetuximab-IONPs, or cetuximab alone. Apoptosis, as indicated by activation of caspase 3 cleavage, was seen only in the U87MGwtEGFR cell line treated with cetuximab-IONPs.

Next, we examined the mechanism of cytotoxicity of the cetuximab-IONPs to GBM neurospheres, focusing on apoptosis and autophagy as putative mechanisms of cell death. In human GBM neurospheres N08-74, N08-30, and N08-1002, none of the treatments induced conversion of LC3B-I to LCB3-II, the hallmark of autophagy (data not shown), suggesting that autophagy is not a likely mode of cell death. In contrast, treatment with cetuximab-IONPs resulted in elevated levels of cleaved caspase 3 and cleaved PARP in GBM neurospheres N08-74, without any cleavage observed in cells treated with free IONPs and cetuximab for 3 hs (Figure 3A, left). After a 14 h incubation, induction of caspase 3 cleavage was even more pronounced (Figure 3A, right). In neurospheres N08-30, treatment with cetuximab-IONPs also resulted in elevated levels of cleaved PARP and cleaved caspase 3. Free IONPs also increased caspase 3 cleavage (Figure 3A, left), most likely due to nonspecific uptake as evidenced in Figure 1C, bottom. On the other hand, in neurospheres N08-1002, both cetuximab-IONPs and cetuximab alone caused apoptosis through cleavage of PARP and caspase 3. Cetuximab-IONPs and cetuximab alone inhibited phosphorylation of Y1068 in EGFR in N08-74 (data not shown). A concomitant decrease in phospho-ERK44/42 levels was also observed in the presence/absence of EGF and FGF (Figure 3B, top) (cetuximab has up to 10-fold higher affinity for EGFR than the EGF and can thus competitively inhibit EGF binding to the receptor [41]). When comparing GBM neurospheres N08-1002 and NHPC, elevated PARP cleavage was observed only in neurospheres treated with cetuximab-IONPs (Figure 3B, bottom). Consistently, U0126 (inhibitor of the ERK pathway) also increased PARP cleavage in cetuximab-IONP-treated cells (data not shown). By subcellular protein fractionation we found that treatment with cetuximab-IONPs for 2 (data not shown) and 5 hs promoted translocation of wtEGFR to the cytoplasmic (data not shown) and predominantly to the cytoskeletal fraction (Figure 3C, 60-fold increase over control as indicated by densitometric analysis). Compared with control, cetuximab-IONP treatment induced increased translocation of wtEGFR to the lysosomes (data not shown), suggesting increased lysosomal degradation of EGFR in the presence cetuximab-IONPs. In U87MG cells, cetuximab-IONP treatment resulted in elevated levels of cleaved caspase 3 only in U87MGwtEGFR cells but not in the parental U87MG cell line with basal level of wtEGFR (Figure 3D). These data highlight the necessity of EGFR for biological activity of the cetuximab-IONPs.

In summary, these data demonstrate that cetuximab-IONPs specifically induce increased apoptosis in GBM neurospheres and cell lines in comparison to NHPC, and promote traslocation of wtEGFR predominantly to the cytoskeletal fraction within cells.

### EGFR profile and characterization of human GSCs *in vitro* and *in vivo*

**Figure 4:**
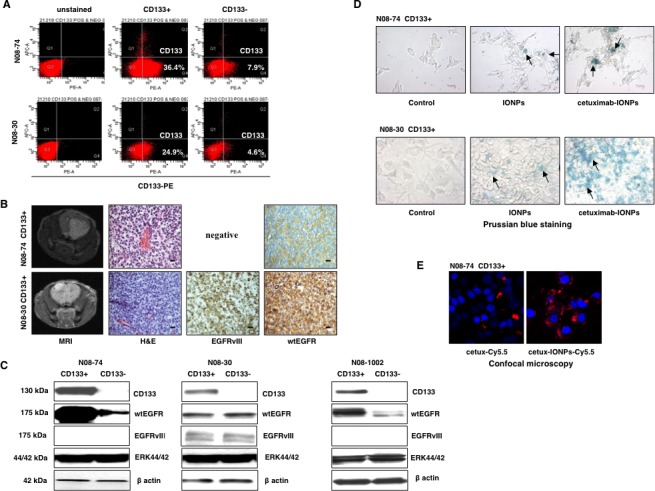
Molecular profile and characterization of human GSCs (A) FACS analysis of human GSCs (CD133-positive) and GBM CD133-negative neurospheres from patients N08-74 and N08-30. (B) MRI, hematoxylin and eosin (H&E), and immunohistochemistry staining of EGFRvIII and wtEGFR in orthotopic human GBM xenografts generated in nude/athymic mice after intracranial implantation of N08-74 and N08-30 GSCs, scale bars, 100 μm (magnification 40x). (C) Expression profile of selected proteins probed by Western blotting in human GSCs (CD133-positive) and GBM CD133-negative neurospheres from patients N08-74, N08-30, and N08-1002, total ERK 44/42 and β-actin were used as internal controls. (D) Prussian blue staining of IONPs and cetuximab-IONPs internalized by N08-74 and N08-30 GSCs (representative slides are shown). After 24 h treatment, neurospheres were allowed to attach to cell culture dish in neurobasal media and stained (nanoparticles are indicated by black arrows, magnification 40x). Cetuximab-IONPs showed maximal uptake in N08-74 and N08-30 GSCs. (E) Confocal microscopy of cetuximab-Cy5.5 and cetuximab-IONPs-Cy5.5 internalized by N08-74 GSCs. After 4 h treatment, GSCs were allowed to attach to culture slides, fixed, and imaged using Zeiss LSM 510 Meta Confocal microscope. Cy5.5, pseudo-colored red; DAPI, pseudo-colored blue (maximum intensity projection, magnification 100x).

Utilizing FACS analysis, we isolated CD133-positive GSCs cells from GBM neurospheres N08-74, N08-30 (Figure 4A), and N08-1002 (data not shown). Renewal capacity and multi-lineage differentiation ability of GSCs were confirmed (data not shown). GSCs (10^4^) from all tested neurospheres (N08-74, N08-30, and N08-1002) were tumorigenic in athymic nude mice and intracranial GBM xenografts were confirmed by both MRI and histological examination. Expression of both wtEGFR and EGFRvIII *in vivo* was demonstrated by immunohistochemistry (Figure 4B). Mice inoculated with N08-30 CD133-negative cells (10x more than GSCs) also developed invasive intracranial tumors at a later point (data not shown). Interestingly, GSCs from neurospheres N08-74 and N08-1002 expressed higher levels of wtEGFR than CD133-negative cells, whereas CD133 status had no effect on wtEGFR and EGFRvIII expression in fractions isolated from neurospheres N08-30 (Figure 4C).

### GSCs bind and internalize cetuximab-IONPs

Internalization of cetuximab-IONPs in CD133-positive N08-74 and N08-30 GSCs *in vitro* was confirmed by Prussian blue staining at 24 hs (Figure 4D). These data confirm again that the cetuximab-IONPs were most efficiently taken up by the N08-74 GSCs and N08-30 GSCs, suggesting improved internalization of cetuximab-IONPs compared to free IONPs. In addition, confocal microscopy of N08-74 GSCs and N08-30 GSCs (data not shown) treated with Cy5.5-conjugated cetuximab or cetuximab-IONPs for 4 hs was performed. The ratio of pseudo-red (Cy5.5) to pseudo-blue (DAPI) signals confirmed more efficient internalization of cetuximab-IONPs-Cy5.5 compared with cetuximab-Cy5.5 alone (7x higher in the former, Figure 4E).

### Cytotoxic effect of cetuximab-IONPs in human GSCs and tumor non-stem cells *in vitro*

**Figure 5:**
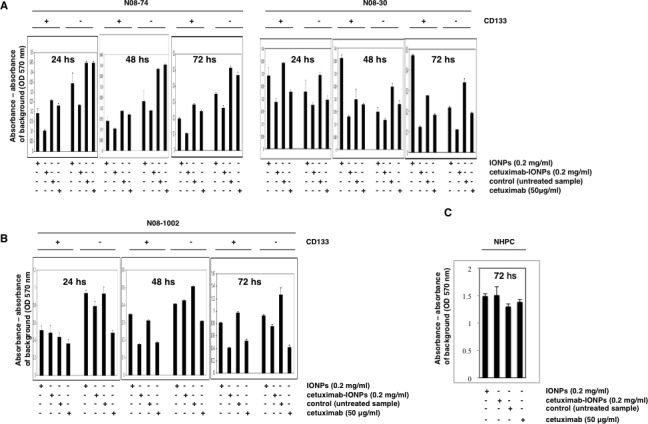
Cytotoxicity of cetuximab-IONPs in human GSCs and GBM CD133-negative cells quantified by MTT assay Human GSCs harvested from neurospheres N08-74, N08-30 (A), and N08-1002 (B) (3×10^4^ cells per well), GBM CD133-negative cells (3×10^4^), and NHPC (C) were treated with free IONPs (0.2 mg/ml), cetuximab-IONPs (0.2 mg/ml), control vehicle, or cetuximab (50 μg/ml). MTT assay was performed after 24, 48, and 72 hs (GSCs and GBM CD133-negative cells) or 72 hs (NHPC). A significant decrease in cell survival was found in all human GSCs treated with the cetuximab-IONPs for 72 hs (*P*<0.001); cetuximab-IONPs also decreased, to a lesser degree, the survival of human GBM CD133-negative cells after 72 hs (*P*<0.001). No cytotoxicity was observed in NHPC cells after 72 hs.

We also investigated the effect of cetuximab-IONPs on the growth of human GSCs. GSCs and GBM CD133-negative cells from N08-74, N08-30 (Figure 5A), and N08-1002 neurospheres with varying levels of EGFR expression (Figure 5B), were treated with free IONPs, cetuximab-IONPs, and cetuximab for 24, 48, and 72 hs. CD133 positivity of GSCs on the day of the experiment was verified by Western blot (data not shown). Cell proliferation and survival assays revealed that cetuximab-IONPs most efficiently decreased survival of GSCs and GBM CD133-negative cells (except N08-1002) after 72 hs (Figure 5A, B) (*P*<0.001). Treatment with cetuximab alone also resulted in decreased cell survival in N08-74, N08-30, and N08-1002 GSCs and in N08-74, N08-30 GBM CD133-negative cells, albeit not as efficient as with the cetuximab-IONPs (Figure 5A, B). None of the treatments affected NHPC treated for 24, 48 (data not shown), and 72 hs (Figure 5C). Increasing concentration of cetuximab to 0.2 and 0.5 mg/ml did not significantly decrease viability of N08-74 GSCs after 72 hs (data not shown). These data confirm a significant cytotoxic effect of cetuximab-IONPs on human GSCs and, to a lesser degree, on the GBM CD133-negative population of cells.

### Cetuximab-IONPs induce apoptosis in human GSCs and tumor non-stem cells

**Figure 6:**
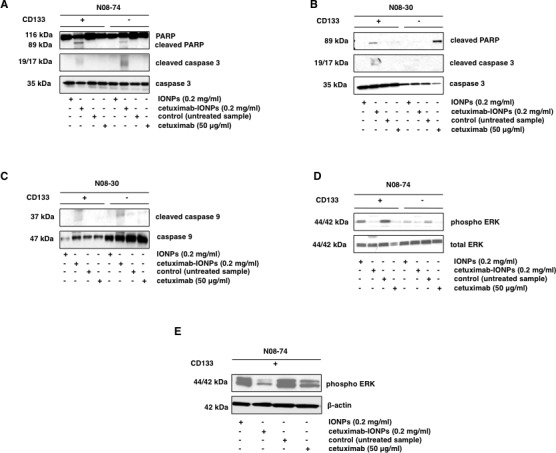
Apoptosis studies and cell signaling in human GSCs after treatment with cetuximab-IONPs GSCs and GBM CD133-negative neurospheres (5×10^5^ cells) from N08-74 (A) and N08-30 (B) were treated with free IONPs, cetuximab-IONPs, and cetuximab for 3 hs and expression of cleaved caspase 3, caspase 3, cleaved PARP, and PARP was determined by Western blot analysis. (C) Expression of cleaved caspase 9, caspase 9 after 3 h treatment in GSCs and GBM CD133-negative neurospheres (5×10^5^ cells) from N08-30. GSCs and GBM CD133-negative cells from neurospheres N08-74 were treated with free IONPs (0.2 mg/ml), cetuximab-IONPs (0.2 mg/ml), or cetuximab (50 μg/ml) for 3 hs and analyzed by Western blotting with phospho-ERK44/42 and total ERK44/42 antibodies (D) or GSCs from N08-74 for 72 hs (E) and analyzed by Western blotting with phospho-ERK44/42 and β-actin antibodies.

Apoptosis and cleavage of caspase 3 was investigated as a mechanism of cell death in GSCs and GBM CD133-negative cells. Both populations from the neurospheres N08-74 and N08-30 were incubated with free IONPs, cetuximab-IONPs, and cetuximab alone. Cetuximab-IONPs induced apoptosis in N08-74 GSCs and GBM CD133-negative cells, as indicated by the presence of cleaved PARP and caspase 3 after treatment for 3 hs (Figure 6A) and cleaved PARP after 6, 8, and 24 hs (data not shown). We did not find any increase in cleaved caspase 3 or PARP after treatment with free IONPs and cetuximab alone. Only cetuximab-IONP-treated N08-30 GSCs underwent apoptosis, as shown by cleaved PARP and caspase 3, whereas cetuximab alone had less of an effect. In CD133-negative GBM cells from N08-30, the opposite was observed: cetuximab alone was more potent than cetuximab-IONPs in inducing cleavage of PARP (Figure 6B).

Drug-treated cancer cells can undergo apoptosis due to activation of intrinsic or extrinsic pathways. We found increased cleavage of caspase 9, a hallmark of activation of intrinsic apoptosis, in N08-30 GSCs and GBM CD133-negative cells treated with cetuximab-IONPs (Figure 6C). Treatment with cetuximab-IONPs and cetuximab alone for 30 min, 1, and 3 hs inhibited phosphorylation of EGFR Y1068 in N08-74 GSCs (data not shown). In addition, significant inhibition of ERK44/42 phosphorylation was observed in N08-74 GSCs, along with a more modest effect in GBM CD133-negative cells treated for 3 hs (Figure 6D). Treatment with cetuximab-IONPs for 72 hs showed more significant inhibition of ERK44/42 phosphorylation than treatment with cetuximab alone in N08-74 (Figure 6E) and N08-30 (data not shown) GSCs. Furthermore, treatment with cetuximab-IONPs and to a lesser extent cetuximab alone dramatically decreased expression of the stem cell marker CD133 in N08-30 GSCs (data not shown) and stem cell markers CD133 and Sox2 in N08-74 GSCs after 3 days (Supplementary Figure S3). Similarly as in GBM neurospheres, subcellular protein fractionation revealed that treatment of GSCs with cetuximab-IONPs for 5 hs promoted translocation of the wtEGFR predominantly to the cytoskeletal fraction (data not shown).

Use of IONPs conjugated to an EGFRvIII antibody (EGFRvIIIAb-IONPs), but not the EGFRvIIIAb alone, induced apoptosis through cleavage of caspase 3 in GSCs and GBM CD133-negative cells from N08-74 (Supplementary Figure S4). These results further support the finding that targeting wtEGFR and the EGFRvIII mutant by EGFR-antibody conjugated IONPs triggers cell death via apoptosis in human GSCs.

### Antitumor effect and animal survival after cetuximab-IONPs CED in multiple orthotopic GBM rodent models

Three different intracranial orthototopic GBM mouse models were used to test the efficacy of cetuximab-IONPs *in vivo*. Animal survival studies were also performed. Human GBM xenograft tumors were established in athymic nude mice with the human GSC-containing neurospheres N08-30, the human GBM U87MGwtEGFR cell line that overexpresses wtEGFR and the CD133 stem cell marker [42], and the GBM LN229wtEGFR cell line that also overexpresses wtEGFR [43]. Athymic nude mice intracranially implanted with N08-30 neurospheres (7 animals per group) and U87MGwtEGFR cells (10 animals per group) were divided into 4 CED treatment groups: cetuximab-IONPs (5μg), free IONPs (5μg,) free cetuximab (5μg), and a control group (HBSS). Mice implanted with LN229wtEGFR (7 animals per group) were divided into 3 CED treatment groups: cetuximab-IONPs (5μg), free cetuximab (5μg), and a control group (HBSS). Before CED, MRI was performed and mice with comparable tumor sizes were randomized into treatment groups.

**Figure 7:**
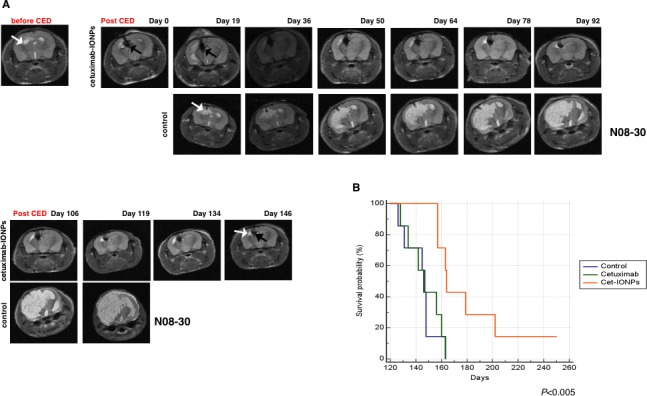
Animal survival studies after CED treatment with cetuximab-IONPs in a human GBM neurosphere model Mice intracranially implanted with EGFR-expressing human GBM neurospheres were subjected to CED with cetuximab-IONPs. (A) T_2_ weighted MRI before CED and days 0, 19, 36, 50, 64, 78, 92, 106, 119, 134, 146 after CED revealed the presence of cetuximab-IONPs (black arrow) and a very small tumor (top and bottom, upper panel, white arrow) in comparison with control mouse (top and bottom, lower panel). (B) Kaplan-Meier survival curve of athymic nude mice intracranially implanted with human GBM neurospheres and CED treated with control, cetuximab, and cetuximab-IONPs. Statistical significance was estimated by log-rank method (*P<*0.005).

In mice implanted with the GSC-containing neurospheres, MRI was performed prior to CED to monitor tumor growth and initiation of CED treatment (Figure 7A). Tumor xenografts derived from the implanted GSC-containing neurospheres had imaging and pathologic characteristics of infiltrating and invasive GBM tumors. After CED of cetuximab-IONPs (day 41 after tumor implantation), MRI on day 0 confirmed T_2_-weighted signal drop and distribution of the cetuximab-IONPs (in the brain 4.79 mm^3^ cetuximab-IONPs is distributed over 1.36 mm^3^ of tumor), T_2_-weighted signal drop correlated with intra- and peri-tumoral cetuximab-IONP distribution (black arrows) (Figure 7A, top and bottom, upper panel). The nanoparticles were still visible on day 146 post CED, revealing delayed clearance of the nanoparticles from the mouse brain. Intracranial xenograft tumors were confirmed by histopathology (Supplementary Figure S5A, left). Prussian blue staining also confirmed intra- and peri-tumoral presence of nanoparticles in the human neurosphere xenografts (Supplementary Figure S5A, right). Immunostaining with wtEGFR and EGFRvIII antibodies confirmed wtEGFR and EGFRvIII expression (Supplementary Figure S5B, left and right). Increased levels of cleaved caspase 3 in tumors from mice treated with cetuximab-IONPs were detected by immunostaining (Supplementary Figure S5C) and Western blotting confirming thus apoptosis *in vivo* (Supplementary Figure S5D). Densitometric analysis of Western blot signals indicated a 27-fold induction of caspase 3 cleavage in cetuximab-IONP-treated xenografts (Supplementary Figure S5E). In contrast, we did not find any increase in cleaved caspase 3 in xenografts treated with free IONPs or cetuximab alone (Supplementary Figure S5D, E).

Overall, mice that received CED of cetuximab-IONPs survived significantly longer (median survival 164 days; *P*<0.005). Median survival time for control mice was 146 days (*P*<0.005) and in animals that underwent CED with cetuximab only, the median survival time was 147 days (*P*<0.005) (Figure 7B). MRI revealed large tumors in control mice and on day 163 post tumor implantation all control mice were dead. In contrast, in the cetuximab-IONP treated group, significantly smaller tumors were detected on days 134, 146 after CED (175 and 187 days post implantation) (Figure 7A). Furthermore, two mice from this treatment group were long-term survivors living over 200 days (Figure 7B).

In the U87MGwtEGFR orthotopic model, CED treatment was performed 5 days after implanting GBM cells. A separate group of 4 mice was sacrificed on day 5 and intracranial xenografts were confirmed by histopathology (Supplementary Figure S6A). After CED, H&E (Supplementary Figure S6B, left) and Prussian blue staining (Supplementary Figure S6B, right) were performed. Immunostaining confirmed wtEGFR expression (Supplementary Figure S6C). Furthermore, potent inhibition of EGFR Y1068 phosphorylation was demonstrated by immunostaining (Supplementary Figure S6D) and Western blot analysis (data not shown), whereas levels of cleaved caspase 3 increased in the intracranial xenografts from mice treated with the cetuximab-IONPs (Supplementary Figure S6E).

**Figure 8:**
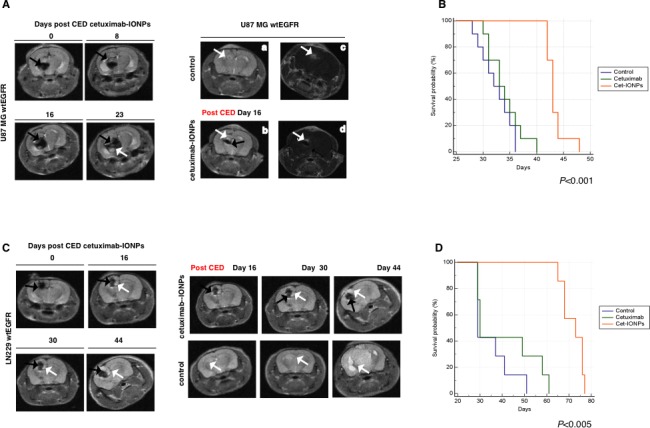
Animal survival studies after CED treatment with cetuximab-IONPs in human GBM U87MGwtEGFR and LN229wtEGFR models Mice implanted with GBM cell lines (EGFR-expressing U87MGwtEGFR and LN229wtEGFR) to form orthotopic human GBM xenografts underwent CED with cetuximab-IONPs. (A left) T_2_ weighted MRI revealed the presence of cetuximab-IONPs (black arrows) and their distribution and dispersion on days 0, 8, 16, and 23, white arrow indicates intracranial U87MGwtEGFR xenograft. (A right) Examples of T_2_ weighted MRI of mice brains showing a GBM xenograft with a bright signal (white arrow) post tumor implantation (day 16) (a); MRI signal drop (black arrow) after cetuximab-IONPs CED (b). Tumor contrast enhancement after administration of gadolinium contrast agent in a control mouse (c) and a mouse treated with cetuximab-IONPs (d). White arrows indicate intracranial xenografts. (B) Kaplan-Meier survival curve of athymic nude mice intracranially implanted with U87MGwtEGFR cells and CED-treated with control, cetuximab, or cetuximab-IONPs. Statistical significance was estimated by log-rank method *(P<*0.001*)*. (C left) T_2_ weighted MRI revealed the presence of cetuximab-IONPs (black arrows) and their distribution and dispersion on days 0, 16, 30, and 44 after CED, white arrow indicates intracranial LN229wtEGFR xenograft. (C right) T_2_ weighted MRI day 16, 30, 44 after CED revealed the presence of cetuximab-IONPs (black arrow) and a small tumor (top panel, white arrow,) in comparison with control mouse (bottom panel, white arrow). (D) Kaplan-Meier survival curve of athymic nude mice intracranially implanted with LN229wtEGFR cells and CED-treated with control, cetuximab and cetuximab-IONPs. Statistical significance was estimated by log-rank method (*P<*0.005).

Additionally, we monitored targeting and the presence of nanoparticles in human GBM xenografts by MRI (Figure 8A). MRI confirmed T_2_-weighted signal drop and distribution of cetuximab-IONPs on day 0 after CED. The T_2_ signal drop increased on days 8, 16, and 23, confirming progressive dispersion of the bioconjugated IONPs into the surrounding brain (Figure 8A, left) as previously described [44]. Dramatic T_2_-weighted signal drop within the xenograft tumor was observed in mice that underwent cetuximab-IONP CED (Figure 8A, right, bottom, black arrow).

In the U87MGwtEGFR model, the median survival time of mice treated with cetuximab-IONPs was 42 days, whereas control mice died in 31 days. Median survival times of animals that underwent CED with free IONPs (data not shown) and cetuximab only were 34 and 33 days, respectively (*P*<0.001) (Figure 8B).

As the final orthotopic model, we employed LN229wtEGFR GBM cells implanted 5 days prior to CED treatment. As in previous models, targeting and the presence of nanoparticles in xenografts was monitored by MRI (Figure 8C). We confirmed dramatic T_2_-weighted signal drop within the xenograft tumor (white arrows) and distribution of cetuximab-IONPs on day 0 after CED. T_2_ signal drop increased on days 16, 30, and 44 confirming dispersion of the bioconjugated IONPs into the surrounding brain (Figure 8C, left, black arrow). On day 49 post tumor implantation (44 post CED), MRI of control mice revealed large tumors (Figure 8C, right, lower panel), whereas significantly smaller tumors were detected in cetuximab-IONP-treated mice (Figure 8C, right, upper panel). Histopathology confirmed the presence of intracranial xenograft tumors that expressed wtEGFR and in intracranial xenografts from mice treated with the cetuximab-IONPs significantly higher caspase 3 cleavage was detected (data not shown).

Most importantly, in the LN229wtEGFR model, the median survival time of mice treated with cetuximab-IONPs was 73 days, two and half time longer than control mice and mice that underwent CED with cetuximab only with median survival times of 29 and 30 days, repectively (*P*<0.005) (Figure 8D). In summary, in all three GBM rodent models tested, CED of cetuximab-IONPs significantly increased survival of animals with human EGFR-expressing orthotopic tumors.

### Intracranial cetuximab-IONP toxicity evaluation

In all 3 rodent GBM models, histopathology examination revealed no signs of toxicity in the surrounding brain tissue where CED was performed. No signs of inflammation, neural cell degeneration, or necrotic cells were found in mice after CED of cetuximab-IONPs (data not shown).

Toxicity studies, performed separately in immunologically competent C57BL/6 mice (without orthotopic xenografts) that underwent cetuximab-IONP CED, provided no evidence of toxicity. After cetuximab-IONP CED, there were no changes in rodent behavior, weight loss, appearance, or neurological signs. Furthermore, MRI showed no signs of hemorrhage, inflammation, or edema on T_2_ weighted-imaging on day 1 (Supplementary Figure S7) and day 7 (data not shown). On histopathologic examination, no toxicity or inflammation was evident. No neural cell degeneration or necrotic cells were found in brains of C57BL/6 mice 14 days and 1 month post CED of cetuximab-IONPs (data not shown).

In conclusion, these data demonstrate that cetuximab-IONPs induce a significant increase in survival of experimental animals implanted with highly tumorigenic GBM xenografts in the absence of any signs of toxicity. We provide evidence that cetuximab-IONPs are feasible for *in vivo* GBM tumor targeting and have a significant antitumor effect.

## DISCUSSION

Unsatisfactory results of standard GBM therapy have resulted in multiple efforts to search for new therapeutic strategies. Proper delivery of therapeutic agents and tumor targeting are important goals that will help overcome obstacles in the treatment of primary malignant brain tumors. GBM tumors which rarely metastasize, and are locally invasive in the brain, represent a potential target for loco-regional delivery strategies, such as CED [45]. CED was developed as a tool for intracerebral drug delivery and its utility has been demonstrated in multiple human clinical trials [46, 47]. CED provides high local concentration of drugs in the brain with relatively uniform distribution permitting subsequent diffusion and further dispersion of drugs into the surrounding brain. The other advantage of CED is the ability to concentrate therapeutic agents into the tumor and surrounding brain tissue and avoid systemic toxicities [48, 49].

The importance of EGFR in the malignant progression of GBM and the effect of EGFR on patient survival has been extensively documented. Heterogeneity of GBM tumors with respect to *in situ EGFR* amplification was previously reported in tumor cells with *EGFR* amplification enriched at the invading margin of tumors [14, 50]. EGFR has been proposed as a prospective GSC marker [51] and targeting of EGFR by small molecules or antibodies is therapeutically relevant [52, 53]. The CD133 marker allows identification of a clinically more aggressive molecular subtype of GBM and is associated with a worse outcome [54]. Newer studies provide evidence that CD133 negative cells can also be tumorigenic in a rodent model, albeit less efficiently as CD133-positive GSCs [55].

IONPs represent a multifunctional clinical platform that can be designed to therapeutically target cancer cells while simultaneously be sensitively imaged by MRI. There have been several attempts to use magnetic nanoparticles to target GBM [33, 56]. IONPs conjugated with RGD have been used for noninvasive monitoring and orthotopic GBM therapy [57]. Other groups studied the effect of curcumin-conjugated nanoparticles on GSCs *in vitro* [58]. The effect of TRAIL conjugated to nanoparticles in GBM models *in vitro* and *in vivo* has also been explored [59]. We have previously described the conjugation of a purified EGFRvIII antibody to amphiphilic triblock copolymer-coated IONPs for the imaging and treatment of GBM [44].

In this study, we are the first to report that IONPs bioconjugated to the chimeric monoclonal EGFR antibody, cetuximab, have a much greater therapeutic effect against GBM than cetuximab alone. In side-by-side comparisons, cetuximab-IONPs were more effective than cetuximab alone and offered significantly increased tumor cell toxicity *in vitro* against human GBM neurospheres, GSCs, and GBM CD133-negative cells expressing various levels of wtEGFR. The significant *in vitro* effect was also observed in human GBM neurospheres, GSCs, and CD133-negative tumor cells which also expressed the EGFRvIII deletion mutant.

Potential mechanisms responsible for the cytotoxic effect of cetuximab-IONPs against different types of GBM cells include increased apoptosis through activation of the intrinsic pathway resulting in elevated cleaved caspase 3 levels. We have also found EGFR signaling alterations, as indicated by significant inhibition of ERK44/42 phosphorylation in GSCs treated with cetuximab-IONPs. We have determined the cetuximab-IONPs bind to both the wtEGFR and the EGFRvIII deletion mutant, are internalized by the tumor cells, and that conjugation with IONPs enhances internalization. We believe when internalized, cetuximab-IONPs accumulate and a pro-apoptotic signal is triggered, as the consequence of EGFR signaling dysregulation, through cleavage of caspase 9, followed by cleavage of caspase 3 that eventually causes cancer cell death. Although the pro-apoptotic effect of EGFR-targeting therapies has been described previously, cetuximab elicited a pro-apoptotic effect only in GBM cell lines with *EGFR* amplification [21]. Moreover, in a subcellular fractionation experiment, we were able to show transport of EGFR to the cytoplasm, predominantly to the cytoskeletal fraction. This suggests increased endocytosis and degradation of EGFR in cells treated with cetuximab-IONPs.

Our data suggest that cetuximab conjugation to IONPs amplifies the biological effect of cetuximab and cetuximab-IONPs are effective against GBM cells with varying levels of EGFR expression. Cetuximab in the cetuximab-IONPs complex performs a dual function: by targeting EGFR, it not only inhibits the EGFR signaling pathway but also facilitates attachment of the IONPs onto the GBM cell surface. The reason for the higher cytotoxicity of cetuximab-IONPs may be greater uptake, compared to free IONPs and cetuximab alone, by GBM cells through receptor-mediated endocytosis. This results in a higher concentration of intracellular IONPs that eventually trigger apoptosis [60].

Furthermore, we have found that treatment of GSCs with cetuximab-IONPs dramatically decreases the expression of the GSC markers CD133 and Sox2, providing further support for targeting of GSCs.

The data presented do show that cetuximab-IONPs have a significant therapeutic effect with 3 different GBM animal models. Cetuximab-IONP CED permitted direct imaging by MRI, revealing intra- and peritumoral localization of the nanoparticles in three orthotopic intracranial rodent GBM models. One of these models is characterized by invasive xenografts grown from patient-derived GSC-containing neurospheres and the two other models with xenografts derived from human GBM cell lines. No evidence of toxicity or inflammation was found in rodents treated by the cetuximab-IONPs. In our previous study, we reported on MRI-guided CED of cetuximab-IONPs and demonstrated its safety and feasibility in healthy canine patients [61]. Present studies involved both mice with xenografts as well as healthy immunocompetent mice. Widespread distribution of the nanoparticles within and adjacent to xenograft tumors was observed in all three models as evidenced by MRI and histopathology. This suggests that cetuximab-IONPs can potentially target the main tumor mass and infiltrating cancer cells residing away from the tumor mass. The survival of athymic nude mice implanted with GBM xenografts was significantly prolonged after CED with the cetuximab-IONPs in all three models.

In summary, we have determined that cetuximab-IONPs bind to both the wtEGFR and the EGFRvIII deletion mutant on human patient-derived GBM cells (including GSCs), inhibit EGFR cell signaling, are internalized by the tumor cells, and promote internalization of the EGFR resulting in enhanced apoptosis. Treatment with cetuximab-IONPs exerted a significant therapeutic effect *in vivo* in 3 different orthotopic GBM mouse models after CED. One of the rodent models developed invasive intracranial human GSC xenografts from a patient with GBM. Cetuximab-IONPs are safe, can be visualized on standard T_2_ weighted MRI, are retained in brain tumors for many weeks with no evidence of toxicity to the surrounding brain. No toxicity to healthy immunocompetent mice was observed after CED of cetuximab-IONPs. We have thus provided a proof of principle that GSCs and GBM tumors can be targeted with cetuximab-conjugated IONPs and established the basis for a future human clinical trial for patients with GBM.

## MATERIALs AND METHODS

### Cell lines

The human GBM cell lines U87MG (ATCC, HTB-14 TM), U87MGwtEGFR (stably overexpressing wtEGFR) [62], and LN229wtEGFR (stably overexpressing wtEGFR) have been described [43]. Expression of wtEGFR was verified by Western blot. Human astrocytes (ScienCell Research Laboratories) and neural human progenitor cells (NHPC) (Lonza) were grown as recommended by suppliers. Normal human adult brain tissue, obtained from patients undergoing epilepsy surgery at Emory University (IRB protocol 642-2005), was cultured in DMEM/F12 (50/50 mix, Cellgro) and 10% FCS (HyClone) in the presence of sodium pyruvate, Penstrep, and L-glutamine (HyClone). Cell lines were used for fewer than 6 months after resuscitation and were routinely tested for *Mycoplasma*, no genotypic authentication was conducted. Each cell line was used in early passage.

### Human GBM neurospheres

Tumor specimens were collected from patients with a histologic diagnosis of GBM (WHO Grade IV astrocytoma). Confirmation of tumor diagnosis and grading was performed by neuro-pathologists at Emory University. Patient tumor specimens (patients # N08-74, N08-1002, N08-30, N09-30, N09-32, N09-33, N09-20, N09-21) were harvested at the time of surgical resection with approval by the Emory University Institutional Review Board (IRB) (protocol 642-2005). Tissues were minced with a scalpel, fragments were digested for 30 min at 37°C with 1 mg/ml collagenase/Dispase (Roche) and separated by Ficoll gradient. GBM neurospheres were cultured in a Neurobasal A-medium with N-2 and B-27 supplements (Invitrogen), 10 ng/ml human recombinant bFGF and 20 ng/ml EGF (both STEMCELL Technologies) at 37°C and 5% CO_2_. Neurospheres were used in early passage for fewer than 6 months after preparation.

### Isolation of human GBM CD133-positive cells using FACS

Neurospheres were dissociated using Accutase (Chemicon), single cell suspensions of 1 - 8 × 10^7^ cells were stained with anti-CD133 /1 (AC133)-phycoerythrin (PE)-coupled antibody (Miltenyi Biotech) and sorted using FACS Vantage SE (Becton Dickinson). In all experiments, human GBM CD133-positive and negative cells were used in early passage.

### Antibody bioconjugation of IONPs

Antibodies used for bioconjugation were: cetuximab (erbitux; Imclone LLC; kindly provided by the pharmacy of Winship Cancer Institute, Emory University), rabbit anti-EGFRvIIIAb (GenScript Corp.), and human IgG (Bethyl Laboratories, Inc., Texas, USA). Antibodies were covalently conjugated to water-soluble IONPs with amphiphilic polymer coating (PEG MW 2000), using the Carboxyl Magnetic Iron Oxide Nanocrystal Conjugation kit (Ocean NanoTech, Arkansas, USA). In this procedure, carboxyl groups on the IONPs were activated in an activation buffer (provided by the manufacturer) containing ethyl dimethylaminoprolyl carbodiimide (EDC) and sulfo-N-hydroxysuccinimide (NHS). Briefly, IONPs were mixed vigorously with the EDC/NHS solution at 25°C for 20 min, the IONPs with activated carboxyl groups (100 μl at 5 mg/ml) were then reacted with cetuximab, EGFRvIII antibody, or human IgG (62.5 μl at 2 mg/ml) at 25°C for 2 hs and the reaction mixtures were stored at 4°C overnight. Unreacted antibody was removed by three rounds of centrifugation using 300K MWCO OMEGA membranes, followed by resuspending IONPs in PBS. Conjugation was visualized using mobility shift in 1% agarose gel. The number of antibody molecules conjugated to IONPs was determined by Bradford assay (Bio-Rad Laboratories).

### Physicochemical characterization of bioconjugates

Hydrodynamic size and zeta potential of the bioconjugated IONPs were measured by the ZetaSizer Nano. Bioconjugates were prepared in a washing buffer (Ocean NanoTech) at 1 mg/ml and sonicated for 5 min. Measurements were performed at 25°C.

### Subcellular Fractionation

GBM neurospheres N08-30 (single cell suspensions, 2×10^6^ cells/6-well plate) were treated with control IgG, IONPs (0.2 mg/ml), cetuximab-IONPs (0.2 mg/ml), and cetuximab (50 μg/ml) for indicated time. Subcellular fractions were separated with the Subcellular Protein Fractionation kit for Cultured cells (Thermo Scientific) according to the manufacturer's instructions.

### Transmission electron microscopy

Single cell suspensions of neurospheres (5×10^4^ cells) were incubated with cetuximab-IONPs, and IONPs (all 0.2 mg/ml) for 24 hs and washed 1x with PBS. After fixing the cells with 2.5% glutaraldehyde solution, TEM was performed with a JEOL JEM-1210 unit (JEOL USA) at 10,000x.

### Prussian blue staining

Single cell suspensions of neurospheres (10^5^ cells/12-well plate) were treated with IONPs (0.2 mg/ml), cetuximab-IONPs (0.2 mg/ml), and cetuximab (50 μg/ml) for 24 hs. We used a concentration 50 μg/ml of cetuximab since it was the same amount we used in conjugation reactions. Cells were then washed 3x with PBS, centrifuged to remove unbound nanoparticles, and replated in the presence of 10% FCS for 12 hs. Cells were fixed with 10% formalin for 20 min at room temperature, washed 3x with PBS, treated with an equal mix of 20% HCl and 10% potassium ferrocyanide (Sigma Aldrich) for 20 min at room temperature, washed with PBS, and imaged using Olympus inverted microscope. Experiments were conducted in triplicates.

### Conjugation of cetuximab-IONPs with Cy5.5 and confocal microscopy

Cy5.5 was conjugated to cetuximab and cetuximab-IONPs with EasyLink APC/Cy5.5 conjugation kit (Abcam, MA, USA). Single-cell suspensions of N08-74 GSCs (2 x10^4^ /8 well chamber slide) were treated with cetuximab-IONPs-Cy5.5 (0.2 mg/ml) and cetuximab-Cy5.5 (50 μg/ml) for 4 hs, fixed, and imaged using Zeiss LSM 510 Meta Confocal microscope. Quantification was performed using Zen 2011 Light Edition. Experiments were conducted in triplicates.

### Cell viability assay (MTT)

Toxicity experiments were performed on GBM neurospheres, GSCs (CD133-positive), CD133-negative tumor cells (3×10^4^ cells), primary culture from normal human brain, NHPC, and U87MGwtEGFR (5×10^3^ cells). Cells were seeded in triplicate on 96-well plates and treated with IONPs, hIgG-IONPs (0.2 mg/ml), cetuximab-IONPs, and cetuximab alone for 24, 48, and 72 hs (neurospheres), 72 hs (normal human brain cells and NHPC), and 144 hs (U87MGwtEGFR cells). Cell viability was determined by an MTT Cell Proliferation Assay kit (Roche). All absorbances were in the linear range and corrected by subtracting the background. Experiments were conducted in triplicates.

### Western blot analysis

GBM neurospheres, GSCs, CD133-negative tumor cells, and human GBM cell lines (single cell suspensions, 3.5×10^5^ cells/6-well plate) were treated with control IgG, IONPs (0.2 mg/ml), hIgG-IONPs (0.2 mg/ml), cetuximab-IONPs (0.2 mg/ml), EGFRvIIIAb-IONPs (0.2 mg/ml), cetuximab (50 μg/ml), and EGFRvIIIAb (50 μg/ml) for indicated time. Cells were centrifuged at 4000 rpm for 5 min, lysed in radioimmunoprecipitation buffer (RIPA, 50 mM Tris, pH 8.0, 150 mM NaCl, 5 mM EDTA, and 1% NP40 with protease and phoshatase inhibitors (Roche)) and protein concentrations were determined using Bio-Rad protein Assay kit (Bio-Rad). Conditions for Western blotting were per manufacturers' recommendations. Primary antibodies used were: mouse anti-β-actin (1:1000, Sigma); rabbit anti-cleaved caspase 3 (1:1000, CellSignaling); rabbit anti-caspase 3 (1:1000, CellSignaling); rabbit anti-cleaved caspase 9 (1:1000, CellSignaling); rabbit anti-caspase 9 (1:1000, CellSignaling); rabbit anti-CD133 (1:1000, CellSignaling); rabbit anti-EGFRvIII (1:1000, GenScript Corp.); mouse anti-phospho-EGFR (Tyr1068, 1:1000, CellSignalling); rabbit anti-wtEGFR (1:1000, Santa Cruz Biotechnology); rabbit anti-phospho-ERK44/42 (1:1000, CellSignaling); rabbit anti-ERK44/42 (1:1000, CellSignaling); mouse anti-GFAP (1:1000, CellSignaling); rabbit anti-Nanog (1:1000, CellSignaling); mouse anti-Nestin (1:500, Abcam); rabbit anti-Olig 2 (1:100, Milipore); rabbit anti-cleaved PARP (1:1000, CellSignaling); rabbit anti-PARP (1:1000, CellSignaling); rabbit anti-Sox2 (1:1000, CellSignaling); mouse anti-β3-tubulin (1:1000, Milipore); and rabbit anti-Vimentin (1:1000, CellSignaling). Immunodetection was with horseradish peroxidase-conjugated secondary antibodies (Dako) and ECL (Hyclone).

### Tumor inoculation and CED

Anesthetized athymic nude mice were placed in a stereotactic instrument and GBM neurospheres N08-30 (1×10^6^ cells resuspended in Neurobasal medium, 5 μl), U87MGwtEGFR and LN229wtEGFR GBM cells (5×10^5^ resuspended in DMEM, 5 μl) in were stereotactically inoculated 1 mm posterior to the coronal suture, 3 mm to the right to the midline and 3 mm below the cortical surface into the right striatum (day 0). On day 5 after tumor implantation (U87MGwtEGFR and LN229wtEGFR) and day 41 (N08-30), mice were randomized into four groups (7 in each group using GBM neurospheres N08-30 and GBM LN229wtEGFR cell line and 10 using GBM U87MGwtEGFR cell line) for CED of: a) untreated control; b) free IONPs; c) cetuximab only; and d) cetuximab-IONPs. The CED infusion apparatus consisted of a hydraulic drive serially connected to a digital syringe pump controller (UltraMicroPumpII, World Precision Instruments, Inc., Sarasota, Florida) and was used according to the manufacturer's instruction. For CED a Hamilton syringe with a 22 gauge, 51 mm needle was used. The needle was inserted through the same skull opening used for tumor cell injection with the pressure 8720 kPa. It was performed with 5 μg total dosage in a 10 μl volume (0.5 mg/ml) at a rate of 0.5 μl/min (20 min of total infusion) in the right striatum (0.624 μg Fe/cetuximab per 1mm^3^ of tumor).

### Intracranial toxicity studies of cetuximab-IONPs

For toxicity studies in C57BL/6-cBrd/cBrd/Cr mice, CED was performed as described above with the 5μg total dosage in a 10 μl volume (0.5 mg/ml) at a rate of 0.5 μl/min (20 min of total infusion) in the right striatum.

### Imaging

Anesthetized mice underwent MRI scanning on a 4.7-T animal MRI scanner using a dedicated mouse coil (Varian Unity). T_2_ weighted fast spin echo sequences with TR/TE=6500/70 ms were typically used for imaging of the tumor and IONPs in the brain.

### Histology

The brains of mice implanted with tumors were harvested and fixed with 10% neutral buffered formalin. Axial sections were made at the level of the needle tract to mark the center of xenografted tumors. Tissue blocks were embedded in paraffin, sectioned, and mounted on slides. Prussian blue staining was performed in a mixture (50/50) of 5% potassium ferrocyanide and 5% HCl for 30 min at 37°C followed by a rinse with distilled water. For immunohistochemistry, slides were incubated with rabbit anti-wtEGFR (1:50, Santa Cruz), rabbit anti-EGFRvIII (1:100, GenScript Corp.), anti-cleaved caspase 3 (1:300,) and anti-phospho-EGFR 1068 (1:3000, both Cell Signaling Technology) antibodies at room temperature for 1 h and detected with biotinylated anti-rabbit secondary antibody in rabbit ABC staining system (Santa Cruz Biotechnology).

### Human orthotopic xenografts

All human orthotopic GBM xenograft studies were performed in six to eight weeks old nude female athymic (nu/nu) mice after approval by the Institutional Animal Care and Use Committee of Emory University.

### Animal survival studies

Athymic nude mice were observed daily to monitor external appearance such as hunching, weight loss, locomotion, and feeding behavior. Animals were sacrificed when neurological deficits such as paresis, seizures, and significant weight loss occurred and mouse brains were fixed in 10% formalin.

### Statistical analysis

Cell cytotoxicity assay data are expressed as an average (+S.D.) of three independent experiments performed in triplicates. The unpaired 2-tailed Student's t test was used to evaluate differences between experimental groups, *P* < 0.05 was considered statistically significant. Animal survival data were analyzed with MedCalc statistical software and presented as Kaplan-Meier plots. Statistical analysis was performed with the log-rank test.

## SUPPLEMENTARY MATERIALS, FIGURES


