# Supplementary material for: Targeted therapy of glioblastoma stem-like cells and tumor non-stem cells using cetuximab-conjugated iron-oxide nanoparticles
Source: Oncotarget. 2015 Mar 12;6(11):8788–806. doi: 10.18632/oncotarget.3554 (PMC4496184; doi:10.18632/oncotarget.3554)
Supplement: Supplementary file 1 [file oncotarget-06-8788-s001.pdf]

Targeted therapy of glioblastoma stem-like cells and tumor non-stem cells using cetuximab-conjugated iron-oxide nanoparticles

Supplementary Material

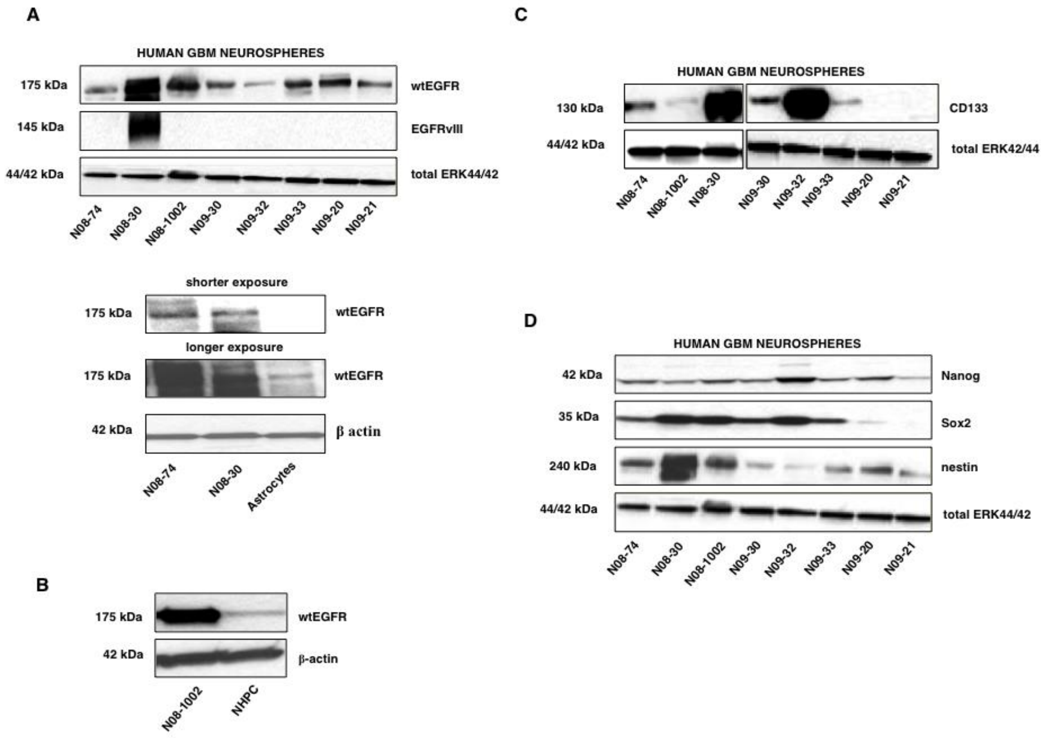

Supplementary Figure 1: Expression of EGFR and molecular profile of GSC-containing human GBM neurospheres. (A) Western blot analysis of EGFR expression in human GBM neurospheres. Only one neurosphere culture (N08-30) is EGFRvIII positive. The lower panel shows that N08-74 and N08-30 neurosphere cultures maintain wtEGFR expression even at late passages (31 and 46, respectively). Normal human astrocytes, used as a control, express low levels of wtEGFR. Short and long exposures are shown. (**B**) Western blot analysis of EGFR expression in human GBM neurospheres in comparison with neural human progenitor cells (NHPC). Western blot analysis of expression of the stem cell markers CD133 **(C)**, Nanog, Sox2, and Nestin **(D)**. Total ERK was used as internal control. In all experiments, neurospheres were used in early passage.

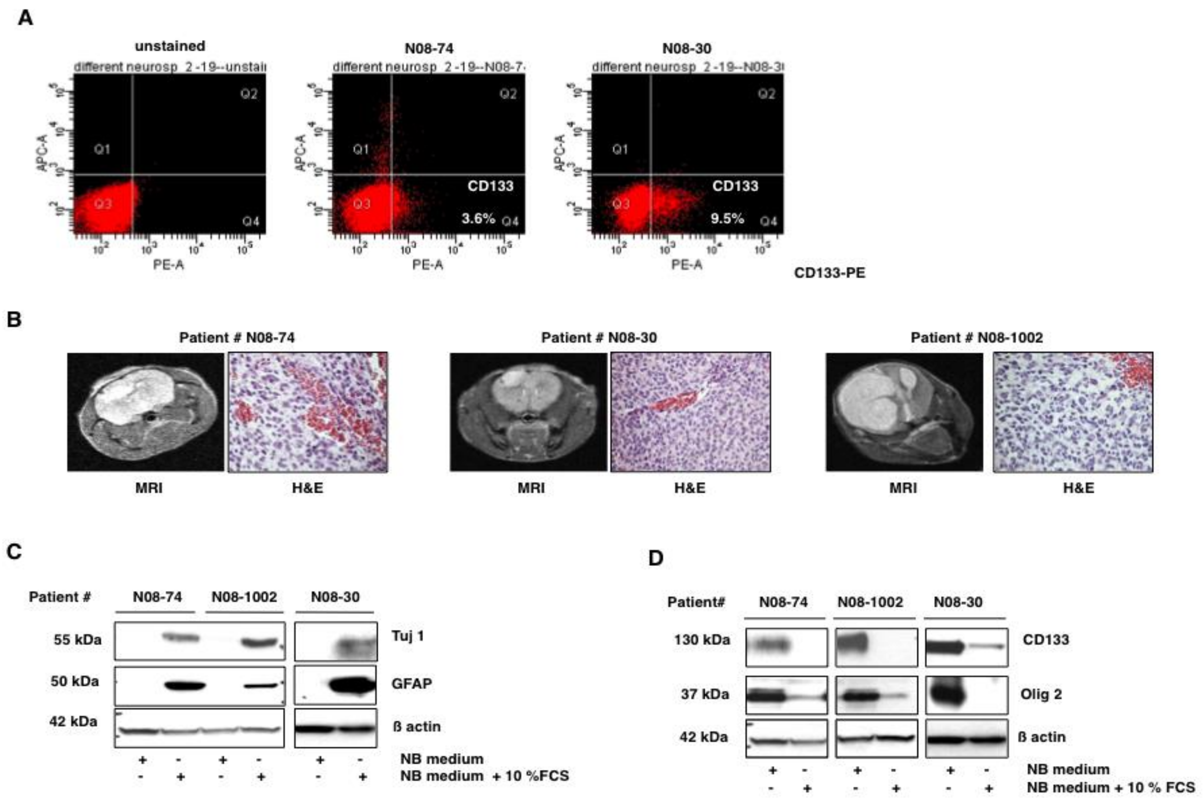

Supplementary Figure 2: Multilineage differentiation and tumorigenicity of human GBM neurospheres. (A) A representative FACS analysis of human GBM neurospheres N08-74 and N08-30 shows different expression levels of the stem cell marker CD133. **(B)** MRI and H&E staining of human GBM xenografts generated in athymic nude mice after intracranial implantation of neurospheres N08-74, N08-30, and N08-1002. **(C and D)** Multilineage differentiation of human GBM neurospheres: Western blotting of neurospheres N08-74, N08-30, and N08-1002, grown in medium with 10% FCS for 11 days, shows multi-lineage differentiation properties as cells became positive for glial (GFAP) and neuronal (Tuj 1) differentiation markers **(C)** whereas expression of the stem cell markers CD133 and Olig 2 was lost or decreased dramatically (**D)**.

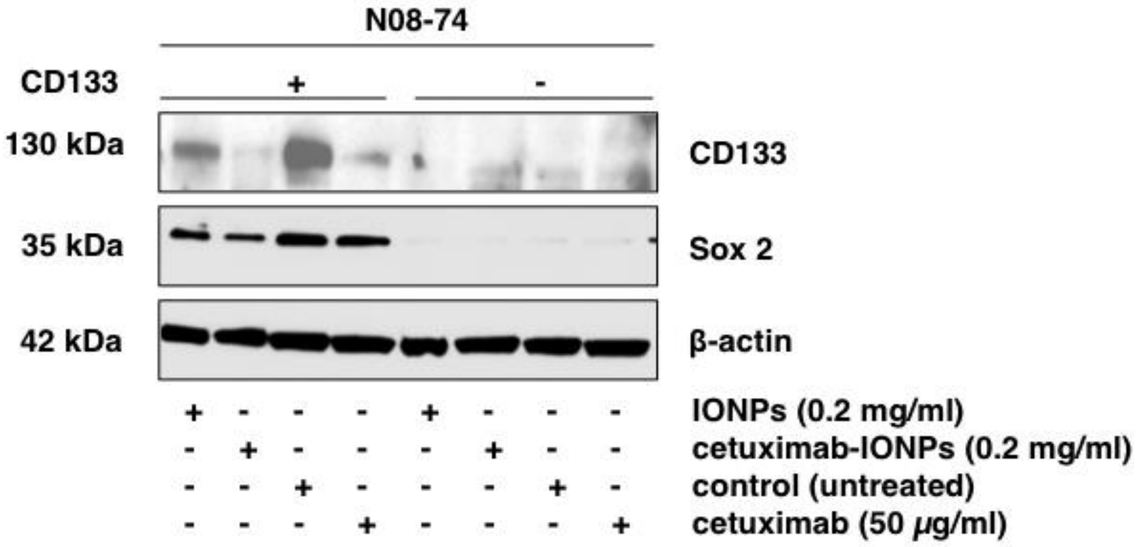

Supplementary Figure 3: Decreased level of stem cell markers in GSCs after treatment with cetuximab-IONPs. GSCs and CD133-negative cells ($5x10^5$ cells) from neurospheres N08-74 were treated with IONPs (0.2 mg/ml), cetuximab-IONPs (0.2 mg/ml), or cetuximab (50 μg/ml) alone for 3 days and analyzed by Western blotting for expression of stem cells markers CD133 and Sox2. Only cetuximab-IONPs decreased expression of CD133 and Sox2 in GSCs.

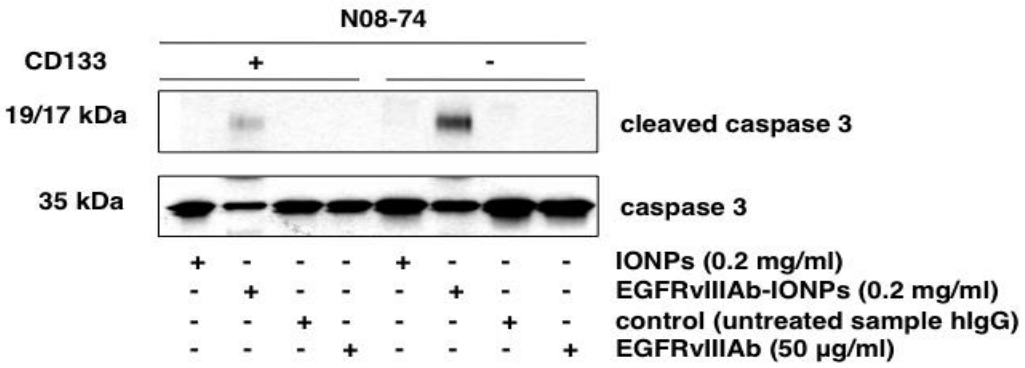

Supplementary Figure 4: Apoptosis in GSCs after treatment with EGFRvIIIAb-IONPs. GSCs and CD133-negative cells ($5x10^5$ cells) from neurospheres N08-74 were treated with IONPs (0.2 mg/ml), EGFRvIIIAb-IONPs (0.2 mg/ml), or EGFRvIIIAb (50 μg/ml) alone for 3 hs and analyzed by Western blotting for expression of cleaved caspase 3 and caspase 3. Only EGFRvIIIAb-IONPs activated caspase 3 cleavage in both GSCs and GBM CD133-negative cells.

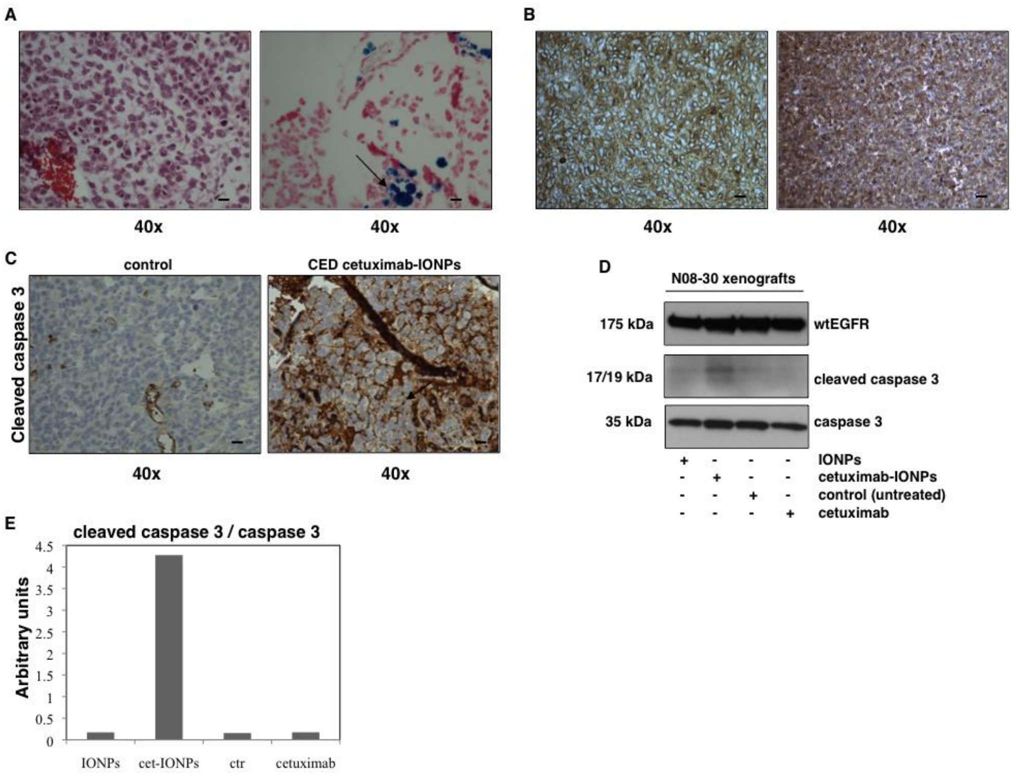

Supplementary Figure 5: Analysis of a representative human GBM xenograft derived from GSC-containing N08-30 neurospheres in the brain of athymic nude mice after CED treatment with cetuximab-IONPs. (A, left) H&E staining confirms the human GBM xenograft (magnification 40x). **(A, right**) Prussian blue staining following CED of cetuximab-IONPs confirms intra-tumoral presence of cetuximab-IONPs (black arrow, magnification 40x). Immunostaining of a human GBM xenograft in an athymic nude mouse for wtEGFR **(B, left)**, EGFRvIII **(B, right)**, and cleaved caspase 3 (control mouse and mouse after CED treatment with the cetuximab-IONPs) (**C)**, (magnification 40x). **(D)** Lysates of xenografts from N08-30 human GBM neurospheres were analyzed by Western blotting for cleaved caspase 3, caspase 3, and wtEGFR expression. Only cetuximab-IONPs induce cleavage of caspase 3. (**E**) Densitometric analysis confirms a significantly higher ratio of cleaved caspase 3/full length caspase 3 in the cetuximab-IONP treated GBM xenografts (P<0.005).

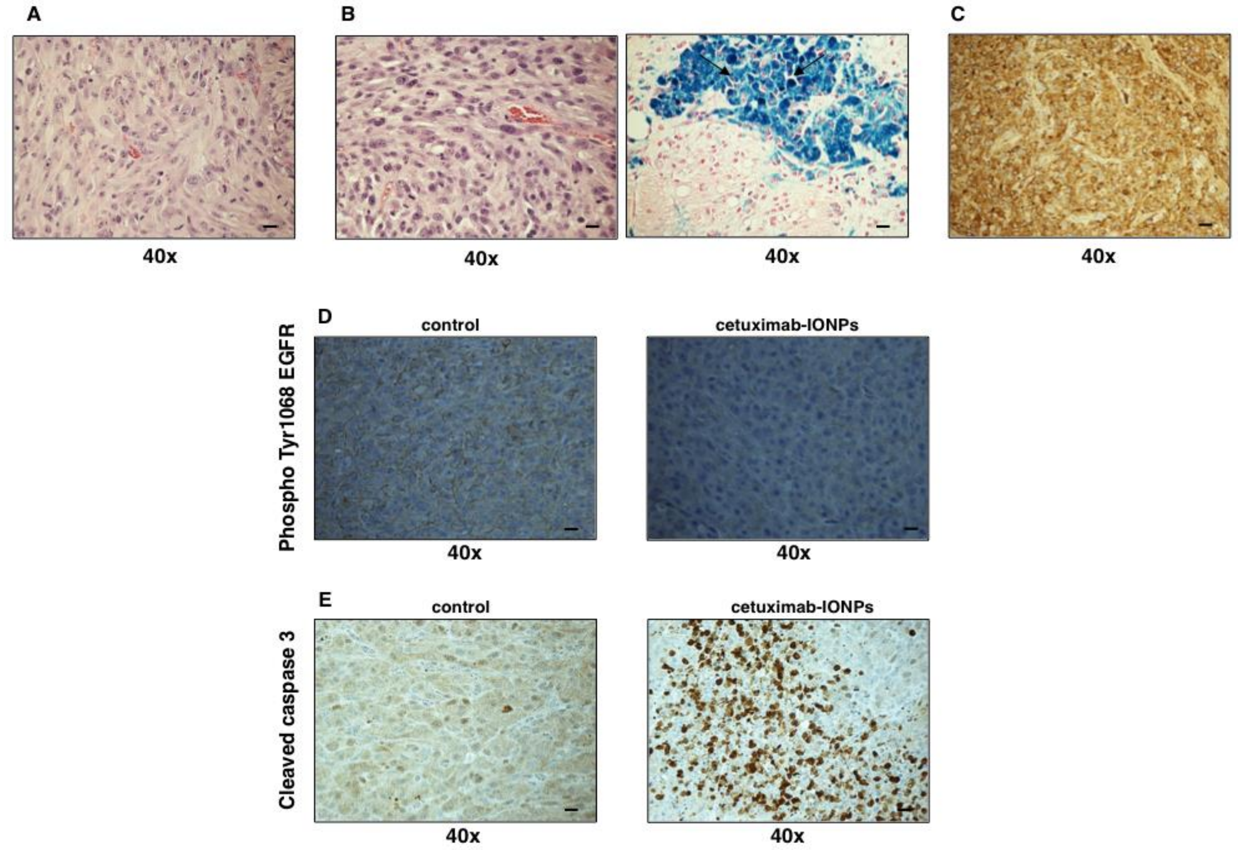

Supplementary Figure 6: Analysis of a representative human GBM xenograft derived from U87MGwtEGFR cells in the brain of athymic nude mice after CED treatment with cetuximab-IONPs. (A) H&E staining confirms the human GBM xenograft 5 days after injection (magnification 40x). **(B, left)** H&E and **(B, right**) Prussian blue staining after CED of cetuximab-IONPs confirms intra-tumoral presence of the cetuximab-IONPs (black arrow, magnification 40x). Immunostaining for wtEGFR **(C)**, phospho-EGFR Y1068 (**D**), and cleaved caspase 3 (**E**) of intracranial human U87MGwtEGFR GBM xenografts in control and cetuximab-IONP CED-treated athymic nude mouse (magnification 40x)**.** Less phosphorylation and increased levels of cleaved caspase 3 were found in cetuximab-IONP treated xenografts.

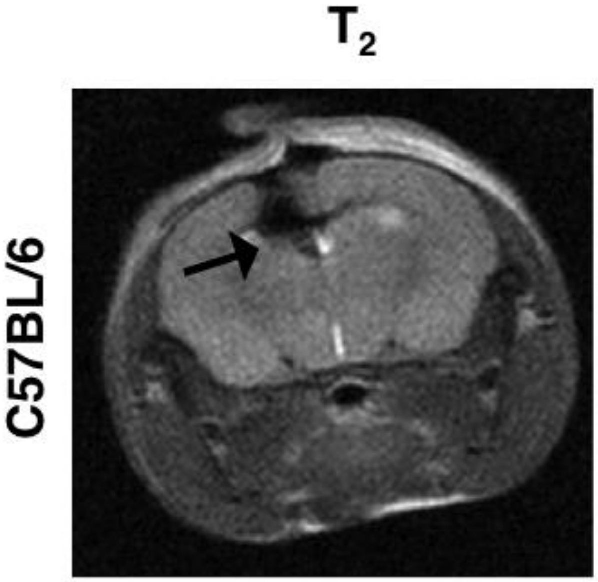

Supplementary Figure 7: Photomicrograph of an immunocompetent mouse brain after CED of cetuximab-IONPs. T_2 weighted MRI after CED of cetuximab-IONPs, day 1 (cetuximab-IONPs, black arrow) shows no hemorrhage in the brain.
